# Transcriptome analysis on the exoskeleton formation in early developmetal stages and reconstruction scenario in growth-moulting in *Litopenaeus vannamei*

**DOI:** 10.1038/s41598-017-01220-6

**Published:** 2017-04-24

**Authors:** Yi Gao, Jiankai Wei, Jianbo Yuan, Xiaojun Zhang, Fuhua Li, Jianhai Xiang

**Affiliations:** 1 Key Laboratory of Experimental Marine Biology, Institute of Oceanology, Chinese Academy of Sciences, Qingdao, 266071 China; 2 Laboratory for Marine Biology and Biotechnology, Qingdao National Laboratory for Marine Science and Technology, Qingdao, 266071 China; 3 Ocean University of China, Qingdao, 266071 China

## Abstract

Exoskeleton construction is an important issue in shrimp. To better understand the molecular mechanism of exoskeleton formation, development and reconstruction, the transcriptome of the entire developmental process in *Litopenaeus vannamei*, including nine early developmental stages and eight adult-moulting stages, was sequenced and analysed using Illumina RNA-seq technology. A total of 117,539 unigenes were obtained, with 41.2% unigenes predicting the full-length coding sequence. Gene Ontology, Clusters of Orthologous Group (COG), the Kyoto Encyclopedia of Genes and Genomes (KEGG) analysis and functional annotation of all unigenes gave a better understanding of the exoskeleton developmental process in *L. vannamei*. As a result, more than six hundred unigenes related to exoskeleton development were identified both in the early developmental stages and adult-moulting. A cascade of sequential expression events of exoskeleton-related genes were summarized, including exoskeleton formation, regulation, synthesis, degradation, mineral absorption/reabsorption, calcification and hardening. This new insight on major transcriptional events provide a deep understanding for exoskeleton formation and reconstruction in *L. vannamei*. In conclusion, this is the first study that characterized the integrated transcriptomic profiles cover the entire exoskeleton development from zygote to adult-moulting in a crustacean, and these findings will serve as significant references for exoskeleton developmental biology and aquaculture research.

## Introduction

As the largest phylum of animals with over a million described species, arthropods make up more than 80% of all known living animal species all over the world^1^. This phylum includes trilobites, chelicerates, myriapods, hexapods, and crustaceans, which inhabit the land, sea and air. The common feature of arthropods is a covering of the exoskeleton^2^. The exoskeleton provides an arthropods shape, water-proofing, locomotion, and can also be very important for growth, development and reproduction^3^. However, this structure also confines metamorphosis, body growth and mating. Thus, arthropods periodically discard their old exoskeletons, meanwhile synthesize and form a new one to match the new body, which is a process referred to as moulting^4, 5^.

In the past century, the structure, chemical composition and mechanical characteristics of the exoskeleton have been widely studied in arthropods^6–10^. Some bio-mimetic composites with high strength and light weight were built and inspired by the exoskeleton^11–14^. The products extracted from the exoskeleton, such as chitin, chitosan and chitosan oligosaccharides have been widely used in the fields of chemical industry and medicine^15, 16^. However, studies on the molecular aspects of exoskeleton development are still scarce, especially analysis of the entire exoskeleton development from zygote to adult. How the exoskeletal regions are constructed and reconstructed to provide their morphological, physiological and mechanical functions is an important issue.

**Figure 1:**
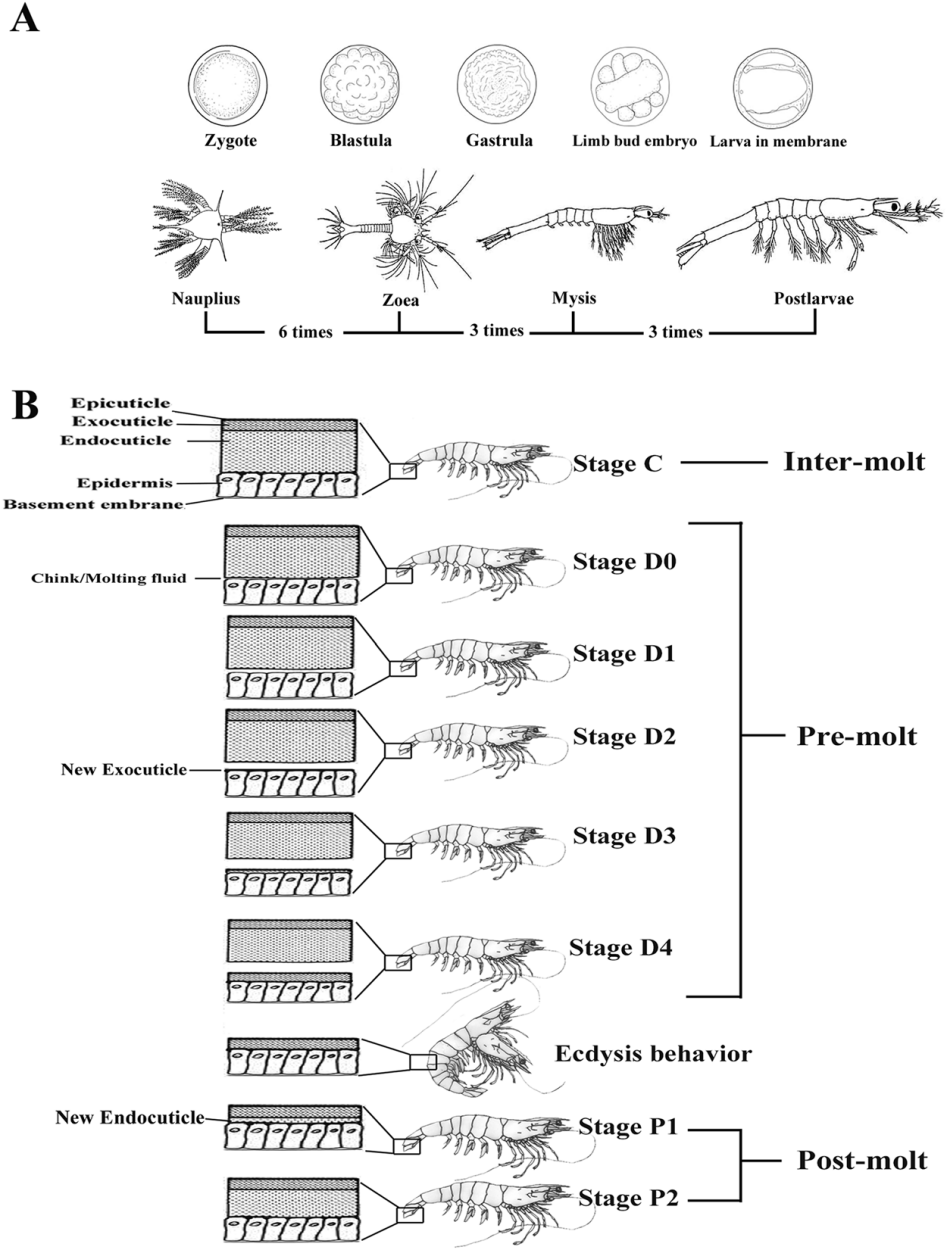
(**A**) Embryonic and larval stages during early development of *L. vannamei*. Morphologies are modified from Wei, *etc*, 2014^19^. The moulting times are labelled under the figure. (**B**) Hierarchical structure of the exoskeleton of *L. vannamei* during the moulting process.

*Litopenaeus vannamei*, one of the most important economic species of decapoda, is the most commonly cultured shrimp species in the world. It is also an ideal model for analysing the exoskeleton development. The exoskeleton alternate is crucial for metamorphosis and growth throughout the life of shrimp. *L. vannamei* experiences 50 moulting periods during a lifetime on average^17^, which is much more than other arthropods; e.g., silkworms (four times), crabs (~eighteen times), and locusts (five times). In early development, *L. vannamei* has a distinctive metamorphosis consisting of nauplius, zoea, mysis and postlarvae^18, 19^ (Fig. 1A). The exoskeleton of shrimp is composed of the polysaccharide chitin, cuticle proteins and mineral deposits. It is a four-layered matrix including an epicuticle, exocuticle, endocuticle and epidermis^20^. According to the appearance of the epidermis, pigmentation, the formation of new setae, and the presence of matrix or internal cones in the setal lumen, the moulting cycle of shrimp can be divided into four recurrent stages: inter-moult, pre-moult, the moment of the moulting behaviour/ecdysis and post-moult^21–23^ (Fig. 1B). For growth, *L. vannamei* need to shed and replace their old exoskeletons and synthesize a new one, and this process is frequently repeated during the life cycle^24^. Failure of moulting in the metamorphosis and mortality of the moulting shrimps are two important reasons for production reduction in aquaculture, which are all closely related to the formation and reconstruction of the exoskeleton. Furthermore, the decapods are a major source of waste shells in the world. It has been reported that world annual bioproduction of waste shells was estimated to be at 6~8 million tonnes^25^. Therefore, many shells urgently need a natural way to degrade. Studies on the development of the exoskeleton in *L. vannamei*, especially in exoskeleton formation, synthesis, degradation, calcification and hardening processes are very valuable for aquaculture, medicine and industry.

In previous studies, molecular research on the exoskeleton in shrimp were mostly concentrated on cloning genes. Several genes related to chitin synthsis^26^, degradation^27^, and mineral absorption^28^ were amplified and sequenced. RNA interference showed that some factors may be associated with exoskeleton reconstruction, such as *CqGAP10* ^29^, *CqGAP65* ^30^, *LvGPS2* ^31^, *CqMIH* ^32^. However, the information collected using these methods is incomplete and provided only a fragmented picture. The molecular mechanisms under exoskeleton development remain poorly understood. The next-generation RNA-sequencing method, which can be used to compare a few genes to the entire transcriptome, could be a vital approach to understand this ontology. This technology has dramatically improved the efficiency and speed of analysis, especially for non-model species, and it is considered to be most powerful tool for dissecting gene networks associated with particular biological and developmental processes at the whole transcriptome level^33–35^.

In this study, we analysed the transcriptomic characterization of *L*. *vannamei* during seventeen dynamic developmental stages from zygote to adult growth-moult by RNA-sequencing method, including nine sequential early developmental stages (zygote, blastula, gastrula, limb bud embryo, larva in membrane, nauplius, zoea, mysis and post-larvae) and eight adult-moulting stages, including inter-moult (C), pre-moult (D0, D1, D2, D3 and D4) and post-moult (P1 and P2). Our analysis underscored the gene expression profiles in dynamic exoskeleton development and deduced transcriptional events involved in the exoskeleton formation, regulation, synthesis, degradation, mineral absorption/reabsorption, calcification and hardening of the exoskeleton. The characterization of the exoskeleton-related genes and pathways will enhanced our understanding of the molecular mechanisms underlying exoskeleton development.

## Result and Discussion

### Sequencing and assembly of the reference transcriptome

As no genome of *L.vannamei* was available, a reference transcriptome was created first to perform digital gene expression profiling analysis. Using the Illumina RNA-seqing technology, two library transcriptomes from adult *L. vannamei* were sequenced, and a total of 11.1 Gb clean bases were obtained (Table 1). Combining our results with the original data previously sequenced by our laboratory (SRR1460493, SRR1460494, SRR1460495, SRR1460504 and SRR1460505) provided a more comprehensive analysis for depicting the transcriptome profiles. As a result, a total of 117,539 unigenes were assembled, with half of the total assembly length (N50) of 1,327 bp and total nucleotides of 83,002,524 bp.

**Table 1:** Summary of transcriptome sequencing from *L. vannamei*.

Sample	Raw Reads	Clean reads	Clean bases	Error(%)	Q20(%)	GC(%)
Lv_A_1	28596204	27067376	2.71 G	0.04	96.82	48.94
Lv_A_2	28596204	27067376	2.71 G	0.05	95.34	48.96
Lv_B_1	29925513	28399611	2.84 G	0.04	96.99	49.00
Lv_B_2	29925513	28399611	2.84 G	0.04	95.66	49.05

1 means left reads, 2 means right reads.

### Functional annotation and classification analysis

For functional annotation of all unigenes, blast alignment against the National Center for Biotechnology Information (NCBI) non-redundant nucleotide/protein sequence database (NR and NT) and Swiss-Prot databases were performed (Supplementary Table S1). By blast searching with a cutoff E-value < 1e^−5^, 32,398 (48.5%) unigenes were found as putative homologues in the NR database, 19,363 (29.0%) unigenes were found as putative homologues in the NT database, and 29,022 (43.4%) were found as putative homologues in the Swiss-Prot database. The E-value and similarity distribution of the best blast hits for unigenes are shown in Supplementary Figure S1A and S1B. The distribution of best hits over species for NR annotation was also analysed (Supplementary Figure S1C). *Daphnia pulex* (10.0%), *Tribolium castaneum* (6.3%) and *Pediculus humanus corporis* (3.9%) possessed the top three maximum unigenes numbers with NR annotation.

**Figure 2:**
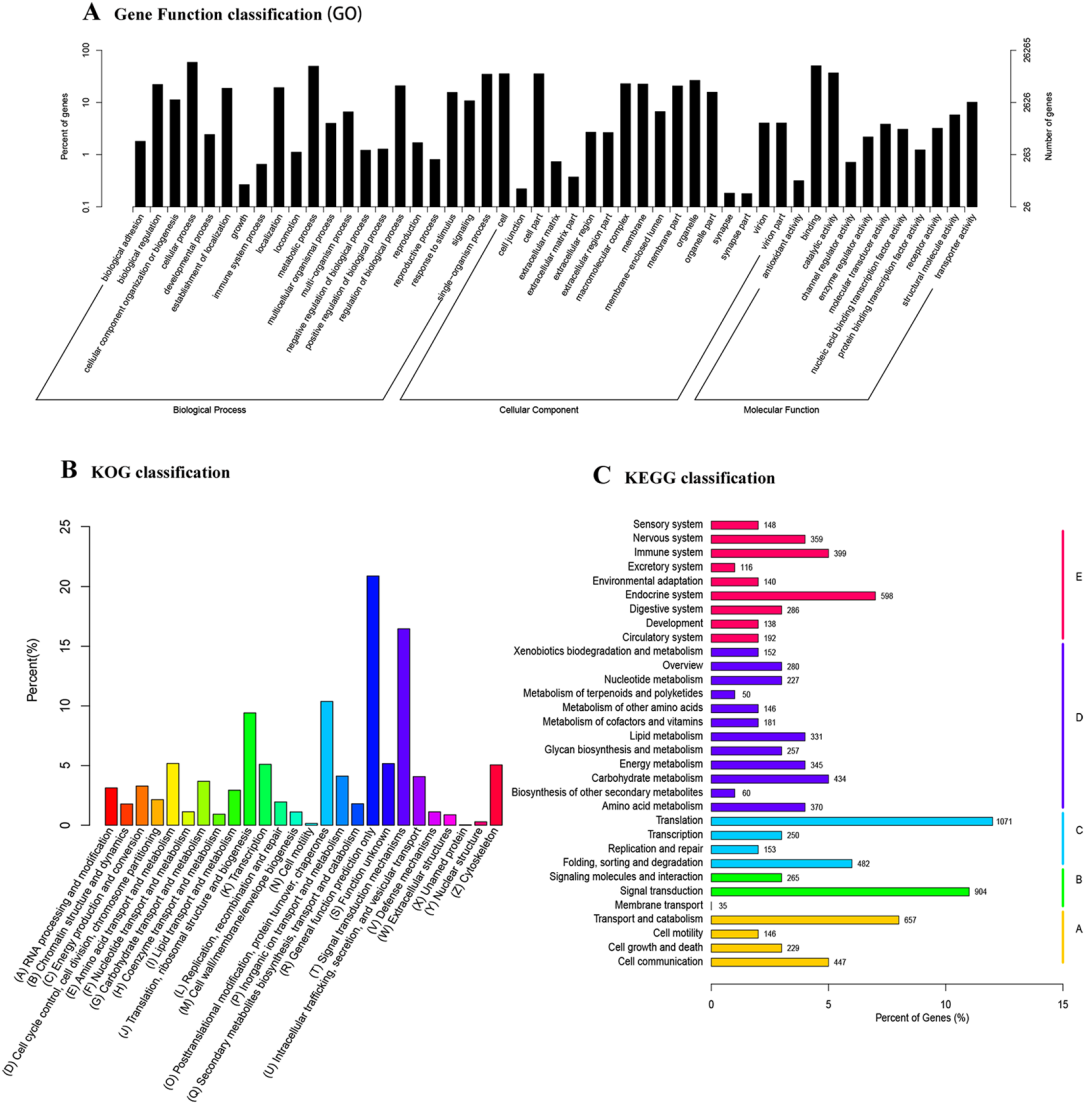
Gene ontology (GO), Clusters of Orthologous Group (COG) and Kyoto Encyclopedia of Genes and Genomes (KEGG) classification of all unigenes. (**A**) The GO classification results summarized in three main GO categories (cellular component, molecular function, and biological process). The x-axis represents the GO ontology. The left y-axis indicates the percentage of unigenes. The right y-axis indicates the number of unigenes. (**B**) COG classification of all unigenes. The columns represent the number of unigenes in each subcategory. (**C**) KEGG classification of all unigenes. The columns represent the number of unigenes in each subcategory.

To acquire complete functional information and pathway-based analysis, Gene Ontology (GO), Clusters of Orthologous Group (COG) and Kyoto Encyclopedia of Genes and Genomes (KEGG) databases were annotated for gene classification and pathway analysis. Through the GO analysis, a total of 158,366 GO terms were associated with all unigenes (Fig. 2A, Supplementary Table S2A). According to the secondary classification of the GO terms, all unigenes were sorted into 49 functional groups that belonged to three main GO categories: biological processes, molecular functions, and cellular components, comprised of 19,445 (34.1%), 21,510 (37.7%), and 16,122 (28.2%) unigenes, respectively (Fig. 2A). In the three categories, the seven subcategories; “cellular process” (15,425 unigenes), “binding” (13,264 unigenes), “metabolic process” (13,030 unigenes), “catalytic activity” (9,673 unigenes), “cell” (9,333 unigenes), “cell part” (9,328 unigenes), “single-organism process” (9,152 unigenes) were included in the top seven most-abundant sub-groups with more than 9000 unigenes.

The COG database is also an important classification system for functional annotation^36^. As for COG classification, 12,368 unigenes (10.52% of all) were classified into 26 functional categories (Fig. 2B, Supplementary Table S2B). The largest group was “general function predicted only” (2,583 unigenes), followed by “Signal transduction mechanisms” (2,036 unigenes), “Posttranslational modification, protein turnover, chaperones” (1,283 unigenes) and “Translation, ribosomal structure and biogenesis” (1,164 unigenes).

Pathway-based analysis helps to further understand the biological pathways of the genes. In the KEGG assignment, 8,498 unigenes were mapped to 259 different pathways (Supplementary Table S2C); a summary of the unigenes assigned to these pathways is shown in Fig. 2C. Of these annotated sequences in KEGG, 28.6% pathways were classified into the pathway related to metabolism, with most of them involved in oxidative phosphorylation (199 unigenes), purine metabolism (192 unigenes), carbon metabolism (169 unigenes), biosynthesis of amino acids (145 unigenes) and pyrimidine metabolism (102 unigenes).

### CDS prediction of the *L. vannamei* transcriptome

As a species lacks genome information, full-length coding sequence (CDS) analysis is very important for molecular research in *L. vannamei* ^37–39^. To better assign full-length gene, the CDSs of all unigenes were analysed by BLASTx and ESTScan. As a result, the CDS of 48,425 unigenes (41.2% of all unigenes) were predicted (Supplementary Table S3). Among them, 19,336 CDSs were extracted from BLASTx against the NR and Swiss-Prot databases. The remaining 29,089 CDSs, with no specific matches to the above databases, were predicted by ESTScan. The distribution of predicted CDS lengths is shown in Table 2, with the full-length coding sequence of nearly half of the unigenes obtained. These results will be of great interest for further analysis in *L. vannamei* as a prelude to functional investigations.

**Table 2:** The length distribution of coding sequence (CDS) predictions.

Gene length(bp)	Number	Percent (%)
51–200	16,677	34.4
201–500	15,278	31.6
501–1000	6,632	13.7
1001–1500	3,921	8.1
1501–2000	2,285	4.7
>2000	3,632	7.5
ALL	48,425	100

For 48,425 of the predicted full-length coding sequences (CDSs), the size distribution can be divided into six species. The total number and the percentage of all genes are presented in the table.

### Digital gene expression profiling analysis of dynamical exoskeleton development

For analysis of the entire exoskeleton developmental process in *L. vannamei*, we sequenced seventeen sequential developmental stages from zygote to adult moult in *L. vannamei*, including nine successive early stages (zygote, blastula, gastrula, limb bud embryo, larva in membrane, nauplius, zoea, mysis and post-larvae) and eight moulting stages, including inter-moult (C), pre-moult (D0, D1, D2, D3 and D4) and post-moult (P1 and P2) by digital gene expression profiling technique.

As a result, seventeen stages produced a total of 233.78 million raw reads with 98.5% Q20 values using the Illumina HiSeq 2500 platform. After removal of low-quality reads, adaptor sequences, duplicate sequences and ambiguous reads, 231.44 million high-quality clean reads (15.73 Gb) remained. Then we mapped the filtered high-quality reads to the reference transcriptome, and 83.11~93% filtered reads were mapped. An overview of the sequencing and assembly results is summarized in Table 3.

**Table 3:** Summary of sequencing and assembly of the transcriptome.

Sample	Raw Reads	Q20(%)	Clean reads	Total mapped	Unigene number	Unigene average length	Unigene N50
Zygo	9074040	98.61	8938449	8249875(92.30%)	27982	1359	2523
blas	11607799	98.45	11464762	10501144(91.59%)	32982	1229	2402
gast	10995688	98.29	10856202	9669177(89.07%)	35979	1166	2323
Lbe	9754060	98.29	9597397	8673620(90.37%)	35526	1174	2298
Lim	8429023	97.83	8335436	7591593(91.08%)	37455	1286	2364
Nauplius	8926413	98.1	8789630	8174251(93.00%)	37531	1337	2348
Zoea	9997945	98.27	9823628	9135914(93.00%)	41711	1299	2264
Mysis	7537908	98.1	7420229	6841843(92.21%)	42655	1319	2239
Postlarvae	8090871	98.1	7961868	7267765(91.28%)	46853	1246	2161
C_1	8462163	98.64	8378139	7205223(86.00%)	50205	1136	2152
C_2	8356671	98.68	8281105	7228674(87.29%)	52156	1112	2109
D0_1	9729632	98.63	9658189	8372908(86.69%)	56217	1063	2059
D0_2	9001963	98.74	8949633	7698406(86.02%)	50493	1133	2147
D1_1	7069133	98.52	7010089	5949698(84.87%)	53411	1102	2085
D1_2	8289817	98.58	8237292	9226254(86.42%)	54246	1085	2097
D2_1	10758027	98.66	10676498	9226254(86.42%)	55917	1060	2059
D2_2	9878904	98.74	9801263	8596987(87.71%)	56264	1068	2070
D3_1	8732484	98.65	8665896	7228584(83.41%)	43427	1189	2256
D3_2	8414766	98.68	8350596	6939910(83.11%)	48107	1139	2160
D4_1	10154005	98.79	10078307	8667790(86.00%)	55468	1064	2066
D4_2	9650580	98.81	9598685	8199145(85.42%)	54727	1082	2077
P1_1	10570861	98.71	10500814	9255363(88.14%)	51858	1117	2126
P1_2	10017393	98.69	9937550	8650085(87.04%)	51350	1122	2133
P2_1	10205898	98.78	10135988	8787490(86.70%)	57581	1047	2030
P2_2	10073944	98.70	9993119	8832160(88.38%)	55285	1071	2068

### Expression profile and differentially expressed gene (DEG) analysis

**Figure 3:**
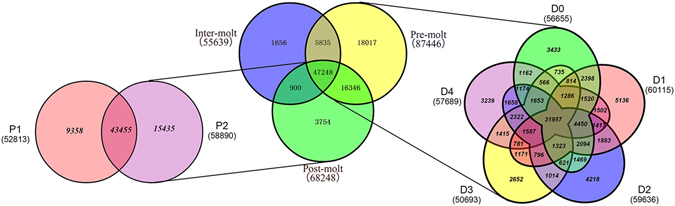
A venn diagram showing the genes expressed in three major stages (inter-moult, pre-moult and post-most) of the moulting process. The details of expressed gene among eight moulting stages are shown.

**Figure 4:**
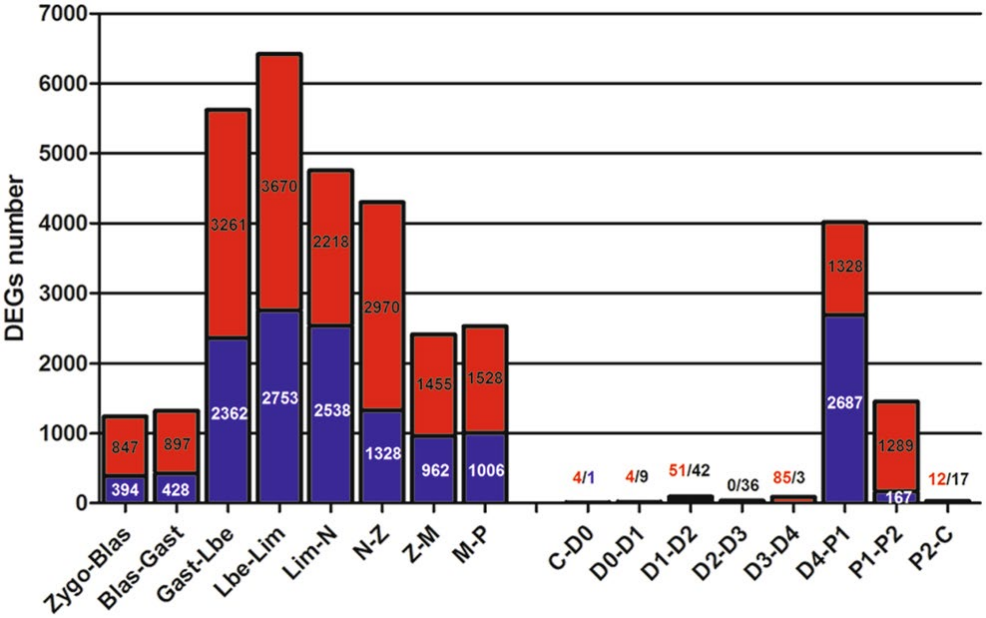
Differentially expressed genes (DEGs) detected between adjacent stages. The numbers of up-regulated and down-regulated genes are revealed by red and blue, respectively.

The expression value of each unigene was estimated by the fragments per kilobase of exon per million fragments mapped (FPKM) method. Among the seventeen developmental stages, the number of expressed genes (FPKM ≥ 0.3) were 27,982 (zygote), 32,982 (blastula), 35,979 (gastrula), 35,526 (limb bud embryo), 37,455 (larva in membrane), 37,531 (nauplius), 41,711 (zoea), 42,655 (mysis), 46,853 (post-larvae), 55,639 (C), 56,655 (D0), 60,115 (D1), 59,636 (D2), 50,693 (D3), 57,689 (D4), 52,813 (P1) and 58,890 (P2). Gene expression during seventeen stages is summarized in Supplementary Table S4 for early developmental stages and Supplementary Table S5 for adult moulting. It showed that the expressed genes were increasing gradually in early development. Also, Venn diagrams showed the number of common and unique genes in different moulting stages (Fig. 3). In this study, differentially expressed gene (DEG) was defined as the fold change of the normalized FPKM values of at least two-fold as well as a false discovery rate (FDR) ≤ 0.001. As a result, a set of 19,809 genes exhibited significant differential expression between two consecutive developmental stages (Fig. 4, Supplementary Table S6).

To validate the sequencing data, eight DEGs were selected for real-time qPCR both in the early development and moulting stages. Expression patterns of the selected genes determined by real-time qPCR (Supplementary Figure S2) had good consistency with the RNA-seq results, which corroborated our results.

### Expression profile of genes related to exoskeleton development

**Figure 5:**
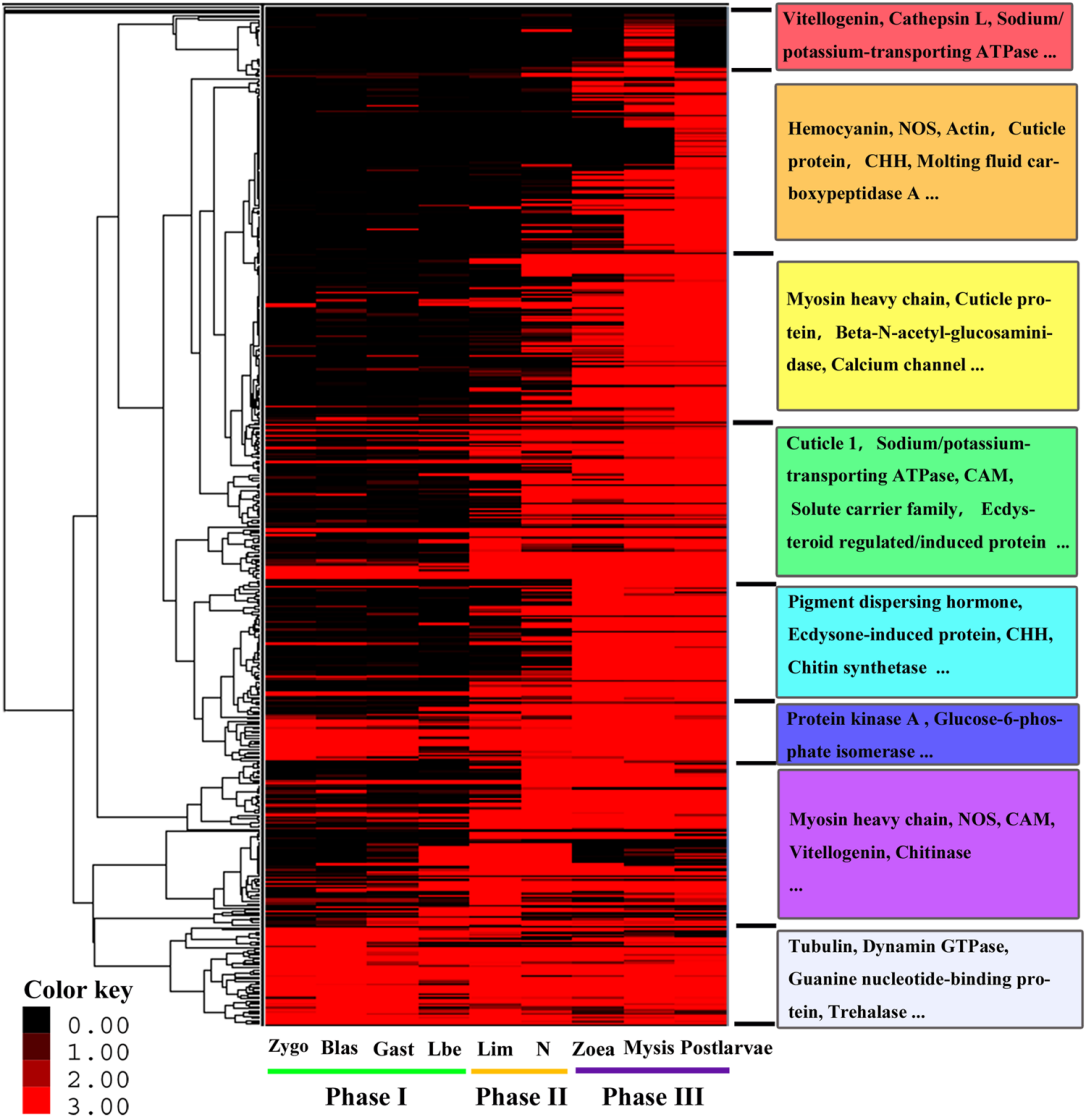
Hierarchical clustering of all exoskeleton-related genes. The clustering indicates similar expression patterns among the developmental stages (x-axis) and among the genes (y-axis). Colours represent the gene expression levels from black (low) to red (high). For each cluster, the major enriched genes are listed.

Based on the functional gene profiling analysis, we summarized the genes related to exoskeleton formation, development and reconstruction from the following perspectives, including: (i) Cuticle protein (cuticle protein, chitin binding protein, structural constituent of cuticle), (ii) Moulting-hormone (moulting-inhibiting hormone, crustacean hyperglycemic hormone *etc*.), (iii) Exoskeleton degradation (chitinase, beta-N-acetyl-glucosaminidase, acetyl-hexosaminidase), (iv) Mineral absorption (solute carrier family, ferritin heavy chain *etc*.), (v) Mineral reabsorption (sodium/potassium-transporting ATPase, clathrin heavy chain *etc*.), (vi) Exoskeleton synthesis (chitin synthetase, Glutamine:Fructose-6-phosphate aminotransferase *etc*.), (vii) Ion channel (calcium channel, potassium channel, chloride channel *etc*.), (viii) Moulting signaling pathway (calmodulin, nitric oxide synthase, moulting defective family member *etc*.) and (ix) Late gene of moulting (actin/tubulin/myosin, hemocyanin *etc*.). As a result, 603 unigenes related to exoskeleton formation, development and reconstruction were identified (Supplementary Table S7). We performed hierarchical clustering of all exoskeleton related genes to examine the similarity and diversity of expression profiles (Fig. 5). The hierarchical clustering generated a global view of the gene expression pattern related to exoskeleton development, indicating that the majority of genes were up-regulated during the middle (lim/nauplius) and late of early developmental stages (zoea/mysis).

### Exoskeleton formation and development during early developmental stages in *L. vannamei*

During the early developmental stages of *L. vannamei*, both morphological and physiological features change dramatically^19^ (Fig. 1A), and this developmental mode provides a unique opportunity to examine exoskeleton formation and development^40, 41^. Based on exoskeleton development, the early developmental stages could be divided into three phases: Phase I, where there is no exoskeleton cover around the body, including zygote, blastula, gastrula, and limb bud embryo; Phase II, the initiation stage of exoskeleton formation, including larva in membrane and nauplius; Phase III, the metamorphosis process, in which there is rapid growth of the exoskeleton. The developmental stages in this phase include zoea, mysis and postlarvae. The transcriptome profile showed a rather different expression pattern among different early stages:

(i) Phase I was significantly enriched in histones, Zinc finger proteins, methyltransferase, and transcription initiative factors, which is related to maternal genes or embryo development; few exoskeleton-related genes were expressed in this phase. Additionally, it appeared that transcriptional repressors were enriched in this stage, and the expression levels of them reduce gradually during the early development. The decrease of the expression levels of repressors meant an active transcription level of metabolism^42^, which was in accordance with our results that gene expression was increasing gradually in early development. (ii) Phase II is the triggering stage of exoskeleton formation, and the specific expressed genes in this phase might be important for exoskeleton formation. Genes of Hox, hormone, transposon-encoded related proteins from larva in membrane to nauplius were significantly enriched, which were related to body plan and hormone regulation^43, 44^; additionally, it appeared that some important exoskeleton-related genes emerged in this phase. For example, the cuticle proteins, which are an important composition of the exoskeleton, were found to be sharply increased to thousands of times in this phase (Table 4). Moreover, moulting-inhibiting hormone (MIH) gene and crustacean hyperglycemic hormone (CHH) gene were triggered their transcription during Phase II. It is now well established that MIH and CHH peptide families serve as key regulators of hormones controlling the moulting process^45, 46^. Their initial expression represents the beginning of moulting-regulation^46^. Therefore, Phase II was the important time node for exoskeleton formation and hormone-regulation, and transcription of the exoskeleton-related genes were triggered in this phase.

**Table 4:** A heatmap showing the expression profile of cuticle proteins (part) during the early development of *L. vannamei*.

Gene ID	NR Description	zygo	blas	gast	lbe	lim	Nauplius	Zoea	Mysis	Postlarvae
c56881_g2	arthrodial cuticle protein AMP8.1	1.59	3.10	0.00	0.00	**24.42**	**3695.25**	1016.29	1194.86	2757.89
c76513_g1	cuticle protein	4.86	2.27	0.83	0.15	**2.89**	**3096.47**	143.43	568.54	525.30
c56881_g1	arthrodial cuticle protein AMP8.1	2.19	1.64	0	0.66	**35.78**	**3009.29**	575.29	923.33	1855.53
c62024_g1	cuticle protein CUT8	0.00	1.24	0.35	0.00	**0.33**	**1479.42**	337.21	1396.92	2408.11
c39382_g1	cuticular protein RR-2 motif 78 precursor	0.65	0.90	0.35	0.00	**1.35**	**948.43**	61.46	86.86	401.31
c65344_g1	cuticle protein	0.66	0.26	0	0.00	**0.00**	**841.99**	166.13	605.69	1011.85
c77367_g1	calcified cuticle protein CP14.1	0.41	0.77	0.35	0.00	**7.17**	**754.50**	457.04	479.15	993.26
c76821_g2	putative cuticle protein (Cpr64Ac)	0.44	1.35	0.00	0.00	**8.84**	**504.28**	0.41	0.51	0.00
c66289_g1	early cuticle protein 6	0.00	0.55	1.89	0.45	**0.00**	**247.32**	139.12	174.46	382.73
c73025_g1	early cuticle protein 6	0.21	0.49	0.00	0.00	**0.00**	**241.66**	29.55	137.76	772.74
c74284_g1	structural constituent of cuticle	0.00	0	0.73	0.20	**0.00**	**233.06**	16.61	25.36	315.62
c66706_g1	structural constituent of cuticle	3.54	113.39	264.43	148.89	**496.93**	**596.02**	21.09	5.46	1.86
c67645_g1	calcified cuticle protein CP8.5	0.00	0.33	0.00	0.00	**0.00**	**97.619**	20.40	10.42	18.36

The gene ID, expression profile (FPKM value) and NR description is depicted. Bold text denotes up-regulation at phase II (larva in membrane and nauplius).

(iii) In the metamorphosis stages (Phase III), a large number of genes related to trypsin, transcription factors, ion channels and receptors, transmembrane receptors, and actin/tubulin/myosin were found to be expressed, which was consistent with some characteristics when converting from larvae to adult, such as larval feeding habits^47^ and formation of the eye^48^. We found that the genes related to chitinase, calcification of the exoskeleton and chitin metabolism occurred at Phase III. Moreover, the downstream hormone-factors under the regulation of MIH and CHH, including ecdysteroid regulated-like protein, ecdysone receptor (ECR), moulting fluid carboxypeptidase, ecdysone-induced proteins *etc*. appeared in these stages. These factors are essential for exoskeleton development and are especially important for synthesis, degradation, regulation, calcification and hardening in the moulting cycle.

In summary, the transcriptome results showed a distinct expression time and profile of developmental genes in the early development of *L. vannamei*. Also, it suggested that the exoskeleton formation was mainly initiate in lim/nauplius stages (phase II). Since factors related to exoskeleton synthesis, degradation, regulation, calcification and hardening were mainly expressed in the phase III, it indicated that an advanced system for exoskeleton development was gradually perfected in late phase.

### Analysis of the exoskeleton degradation in the moulting

**Figure 6:**
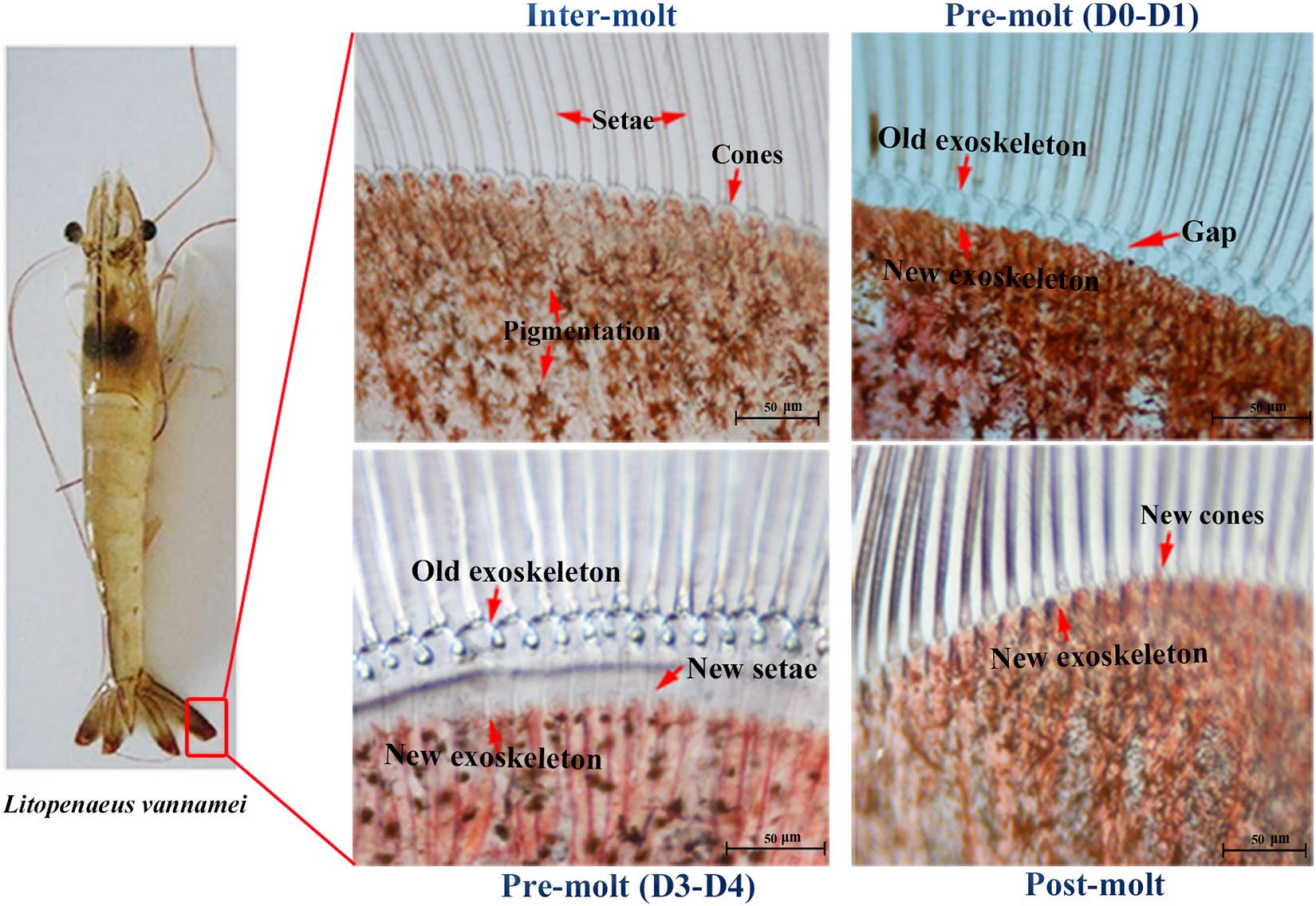
Morphological changes of shrimp uropods during moulting stages under a light microscope.

In shrimp, the exoskeleton reconstruction is a stepwise process that has several landmark events; i.e., apolysis (the separation of the cuticle from the epidermis), new exoskeleton synthesis and shedding of the old exoskeleton^49–51^. As the major component that breaks down the glycosidic bonds in chitin and partially digested old exoskeleton, chitinase was the major downstream factors of ecdysone to break off the old exoskeleton^50^. Forty-four unigenes related to chitinase were identified in the moulting cycle of *L. vannamei* (Supplementary Table S7). It revealed that these genes corresponding to chitin degradation were preferentially expressed during the D0-D1 and period D4. The activity of chitinase in the early pre-moult phase might be associated with the separation of the old and new exoskeleton (Fig. 6). Also, during ecdysis behaviour, the shrimp needed to digest the old exoskeleton for exfoliation. This might be the reason for obvious up-regulation of chitin degradation related genes in period D4. After moulting (P1), the expression of chitin degradation genes reaches the minimum. Similar results regarding the activity of chitinase during the post-moult phase were found in the Antarctic krill *Euphausia superb* ^52^, the fiddler crab *Uca pugilator* ^53^ and the whiteleg shrimp^26^, which have up-regulated expression of chitinase in the pre-moult phase.

These results provided significant clues for a better understanding of the regulatory mechanisms under exoskeleton degradation in *L. vannamei*, and revealed that the D0-D1 and D4 periods were the important time nodes for exoskeleton degradation and indicated some key genes that have potential value for scientific research and applications.

It is known that world annual bioproduction of waste chitin shells is enormous, and the traditional method for extracting chemicals from waste shells is destructive, wasteful and expensive^25^. For example, to produce 1 kilogram of chitosan from shrimp shells, more than 1 tonne of water is need and as well as a large amount of 40% sodium hydroxide solution. Thus, developing a natural way to degrade the waste shells will be promising and necessary^54, 55^. From our results, several chitin degradation-related genes with high expression in the moulting process could be targeted candidates. For instance, the FPKM values of the c78849_g2 (*chitinase*) increased more than 2,500 at the D3-D4 period, which might be an important factor for chitin degradation. In future practical applications, by a proper combination of practical manufacturing technology, such as nano-material and film-material^56^, a novel method for degradation of waste shells could have great potential.

### Analysis of chitin synthesis in *L. vannamei*

**Figure 7:**
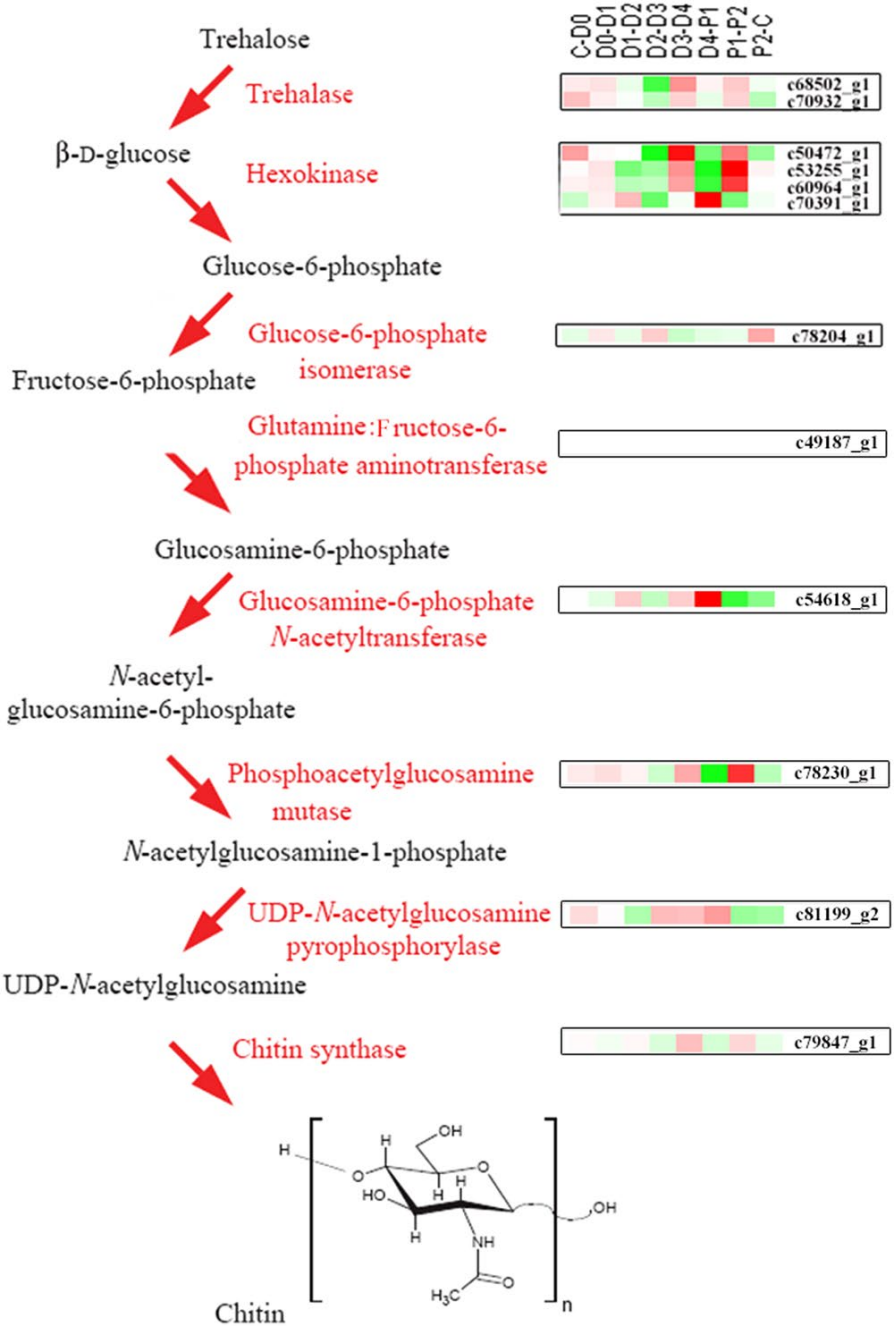
Expression profile of chitin synthesis pathways using the insect analogy. It displays expression patterns for all chitin synthesis factors in each comparison. The colour key represents FPKM normalized log_2_ transformed counts. Red represents up-regulated expression and green represents down-egulated expression. The diagrammatic representation is based on previous studies in insects^57^.

Chitin, a polymer of N-acetyl-β-D-glucosamine, is the major component of the shrimp exoskeleton^26^. As a framework material, chitin supports the exoskeleton of shrimp. Moreover, the moulting of shrimp is strictly dependent on the capability to synthesize chitin^57^. However, knowledge of its biosynthesis is still fragmentary in crustaceans, and is far behind this analysis in insects^58–60^. Based on the transcriptome results, we searched for chitin synthesis pathways be referring to the insect analysis^57^. Identifying the pathways of chitin synthesis in the *L. vannamei* analogy of the insect seemed to be considerable conserved (Fig. 7). Expression profile analysis showed that an up-regulated expression trend was found in the chitin pathway during the D4 and P2 period. Among the pathways, the expression of a chitin synthetase gene (c79847_g1), which encodes the key enzyme in chitin synthesis, significantly increased expression to the peak in the D4 and P2 periods. It is well known that partial digestion of the exoskeleton in the pre-moult phase is accompanied by the synthesis of new underlying exocuticle and epicuticle^61, 62^. Therefore, this up-regulation of the chitin synthesis pathway, together with the functional annotation of the physiological process, suggested that the synthesis of the new exoskeleton was mainly occurring in the D4 and P2 periods. These results provide a vital resource for further research into chitin synthesis in shrimp. Moreover, the temporal stage-related scheduling of the expression of the factors may serve as markers in future studies for monitoring the chitin synthesis process in *L. vannamei*.

### Mineral absorption/reabsorption and the exoskeleton hardening process during moulting

There is mineralization in shrimp exoskeleton, typically calcium carbonate, which gives the exoskeleton mechanical rigidity^2^. It is well known that there is a structure called gastrolith in crayfish that can collapse into the stomach and dissolve to promote minerals for the new exoskeleton^63, 64^. However, there is no such structure that has been reported in shrimp, thus the mineral for moulting was supposed to be absorbed from seawater or reabsorbed in *L. vannamei* ^65^. The annotated transcriptome was searched for mineral absorption and reabsorption encoding genes using the KEGG database. As a result, ko04978 “Mineral absorption” and ko04961 “Endocrine and other factor-regulated calcium reabsorption” were analysed, and the expression levels of the members are summarized in Supplementary Table S8. The results showed that mineral absorption was mainly occurring in the D1 and D4 period, and reabsorption was mainly occurring in the D2 and D4 period. During the early stage of pre-moult, minerals derived from the degradation of the old exoskeleton are not available^66, 67^, hence the mineral absorption (D1) was earlier than the reabsorption of minerals (D2). In addition, they all followed the initial degradation in D0-D1, which was thought to be the onset of moulting.

**Figure 8:**
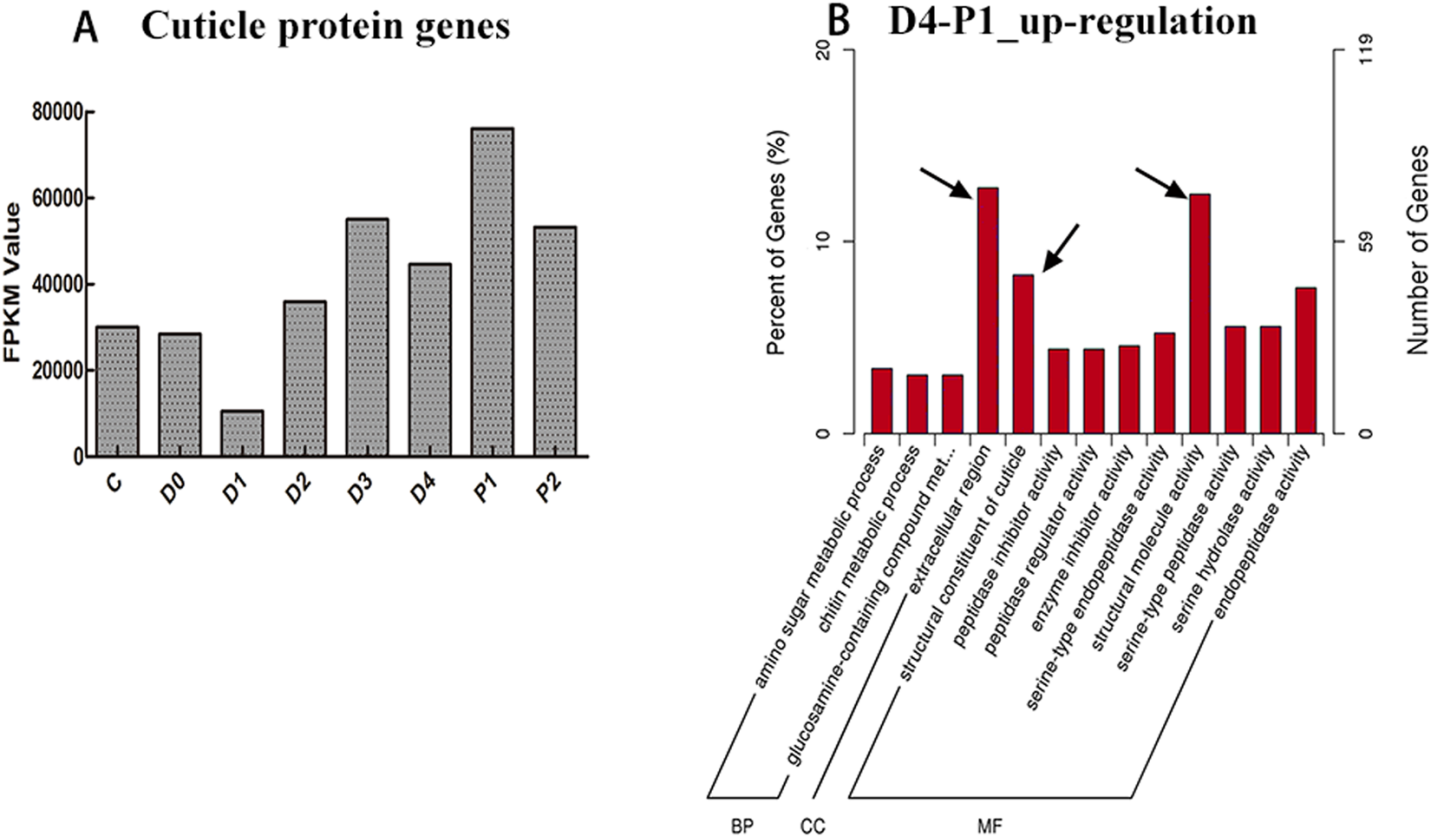
Expression profiles of cuticle proteins and GO enrichment of up-regulated differentially expressed genes (DEGs) in D4-P1. (**A**) Expression profiles of cuticle protein genes. Row axis represents the developmental stages. Columns represent the total FPKM value of genes. (**B**) The GO terms of up-regulated DEGs in D4-P1. The arrows indicate the terms related to exoskeleton. Extracellular region (GO:0005576) with 76 DEGs are dominant in all categories. Structural molecule activity (GO:0005198) and Structural constituent of cuticle (GO:0042302) consisted of 74 and 49 DEGs that were dominant in categories of molecular function. The x-axis represents the name of GO subcategories. The left y-axis indicates the percent of genes. The right y-axis indicates the number of DEGs expressed in a given sub-category. The English in this document has been checked by native English-language editors. And the logical presentation of ideas and the structure of the paper were also checked during the editing process. The edit was performed by professional editors at American Journal Experts (www.aje.com), which is recommended by editorial staffs of Scientific Reports.

As the protein embedded in the chitin, the physical properties of the exoskeleton are determined largely by the cuticle proteins^51^, especially the calcified cuticle proteins, which could support the hardness of the exoskeleton^68^. It was reported that proteins with the cuticle_1 domain were associated with exoskeleton hardening in decapod crustaceans^69, 70^. In terms of RNA-seq results, a total of seventy-three cuticle protein related genes were identified from our transcriptome data (Supplementary Table S7), and the expression pattern on the whole is summarized in Fig. 8A. We found that the expression of cuticle proteins accumulated at a peak after ecdysis (P1). GO analysis also showed an obvious up-regulation of GO terms related to the structure (“GO:0005576” extracellular region, “GO:0042302” structural constituent of cuticle, “GO:0005198” structural molecule activity) between D4 and P1 (Fig. 8B). The abundant expression of cuticle proteins and structure related terms in P1 was considered to be the method for supporting and hardening the soft exoskeleton after moulting.

In summary, a coordination of exoskeleton degradation, chitin synthesis and exoskeleton hardening combined with mineral absorption and reabsorption were collectively occurring during moulting with a programmed procedure and strict control that beginning with the initial degradation in D0-D1, then mineral absorption in D1, followed by mineral reabsorption in D2 for mineral reserve. Then the synthesis of new chitin (D4) occurred, which was the main component of the outer layer-epicuticle^71^. The cuticle proteins, the important parts of the inner three layers^66^, were produced in P1. Finally, the new chitin continuously synthesized (P2) for a complete exoskeleton. The transcriptome analysis on the exoskeleton reconstruction scenario in growth-moulting was summaried in Table 5. This high degree of order is the key element in the many occurrences of moulting repeated during the life cycle of shrimp.

**Table 5:** A summary of transcriptional events applicable to the early development and moulting cycle in *L. vannamei*

Stage	Transcriptional events
D0-D1	Initiative transcription for exoskeleton degradation
D1	Mineral absorption
D2	Mineral reabsorption
D4	Important period for old exoskeleton shed, new chitin synthesis; mineral absorption/reabsorption
P1	Period for new cuticle protein synthesis
P2	Period for new chitin synthesis

## Conclusion

This paper presents the first transcriptome analysis to cover the entire and dynamic exoskeleton formation, development and reconstruction in shrimp, including continuous early development stages and the entire moulting cycle. It establishes a transcriptional scenario for exoskeleton development and deduces the transcriptional events involved from the several perspective, including exoskeleton formation, regulation, synthesis, degradation, mineral absorption/reabsorption, calcification and hardening, *etc*. Moreover, it provides useful insights into the molecular processes underlying these transcriptional events. It indicates that a complex regulatory network of genes is involved in exoskeleton formation, development and construction in *L. vannamei*. Moreover, these genes show a strong coordination with a high degree of order during development. These results will provide molecular evidence for enhancing our understanding of exoskeleton development in shrimp and may possibly serve as a blueprint for other arthropods in future research.

## Methods

### Sample collection and RNA isolation

Healthy adult *Litopenaeus vannamei* with an average body length of 14–16 cm were collected from culture pond of the laboratory for *de novo* sequencing. They were from the same generation and had cultured more than half year to minimize the smallest effects by the environment. Two *de novo* libraries were constructed with mixed samples.

The early developmental samples were collected from a farm in Wenchang, Hainan, China. A total of nine samples, including zygote, cell, blastula, gastrula, limb bud embryo, larva in membrane, nauplius, zoea, mysis and postlarvae, were collected as the pool based on both morphological classification and physiological characters (Fig. 1A). They were reared in a 25 m^3^ indoor ponds with seawater at 31 °C, salinity of 2.5%. Embryos and larvae were collected with screen mesh when 90% of the population had reached the objective stage.

The samples at different moulting stages were collected from the whole individual shrimp with an average body length of 10–12 cm, which were cultured in the pond of our laboratory (seawater at 25 °C, salinity of 3%.). The moulting stages including eight periods, inter-moult (C), pre-moult (D0, D1, D2, D3 and D4) and post-moult (P1 and P2) were separately collected, identified by the formation of new setae, the appearance of new epidermis, and the presence of matrix or internal cones in the setal lumen^21^. Each stage used one individual in order to get the accurate moulting-stage, and each stage was analysed with biological replicates to ensure the accuracy. These materials were immediately frozen in liquid nitrogen and kept at −80 °C. All procedures involving animals were performed per the institutional ethic committee guidelines of Institute of Oceanology Chinese Academy of Sciences (IOCAS).

Total RNA was extracted from samples using the Trans-up RNA reagent according to the manufacturer’s protocol. DNase digestion was completed during RNA purification using the RNase Free DNase Set (QIAGEN). The yield, purity and integrity of total RNA were assessed by electrophoresis in 1% agarose gels and quantified with a NanoDrop 2000 spectrophotometer (Thermo Scientific, USA) and Agilent 2100 bioanalyser.

### Library construction, sequencing and assembly

The RNA-sequencing libraries were prepared according to Illumina’s RNA sample preparation protocol. Magnetic beads with oligo dT were used to isolate poly (A) mRNA from total RNA. Then, fragmentation buffer was added for interrupting mRNA to short fragments. These short fragments were used as templates for random hexamer-primed synthesis of first-strand cDNA. The second-strand cDNA was synthesized using Buffer, RNase H, dNTPs and DNA polymerase I. A paired-end library was synthesized using the Genomic Sample Preparation Kit (Illumina). Short fragments were purified with a QiaQuick PCR extraction kit (Qiagen, Germany). For *de novo* sequencing, the constructed sequencing library was sequenced using Illumina HiSeq 2500 under the 2 × 100 bp paired-end model. For digital gene expression profiling, agarose gel electrophoresis was used to select the fragments with about 50 bp in size. Then the short fragments were connected with sequencing adapters. Finally, the libraries were sequenced using Illumina HiSeq™ 2500 (Novogene, Beijing).

Raw image data was transformed into raw reads by base calling, and stored in fastq format. After removing empty reads, adaptor sequences and low quality sequences, the raw reads were transformed into clean reads. The quality control of the reads was checked with FASTQC (http://www.bioinformatics.babraham.ac.uk/projects/fastqc/). A reference transcriptome was assembled using a subset of reads from seven libraries, which were combined with the original data previously sequenced by our laboratory (SRR1460493, SRR1460494, SRR1460495, SRR1460504 and SRR1460505). The *de novo* transcriptome was assembled using Trinity (version trinityrnaseq_r2013_08_14) to obtain transcripts with min_kmer_cov set to 2 and all other parameters set to default^72^. Unigenes were obtained after redundant transcripts were removed by clustering in Tgicl. Then, reads from each of the development stages were mapped back to the reference transcriptome using the Bowtie2 aligner (parameters: mismatch 0) and converted into fragments per kilobase of transcript per million base pairs sequenced (FPKM)^73^.

### Gene annotation, classification and CDS prediction

Gene function and classification were analysed based on searches against the following databases: National Center for Biotechnology Information (NCBI) non-redundant nucleotide/protein sequence database (NR and NT), SwissProt database, Gene Ontology (GO), Clusters of orthologous groups of proteins (COG), Kyoto Encyclopedia of Genes and Genomes (KEGG) database. The software and methods for annotation and classification were: NCBI blast 2.2.28 + for NR, NT and SwissProt annotation with the E-value threshold was <1e^−5^. Blast2GO (version 3.0) (https://www.blast2go.com/) was used for GO annotation with E-value < 1e^−6^ ^74^; WEGO was used to categorize GO term to view the distribution^75^. KAAS (http://www.genome.jp/tools/kaas/) and KEGG Automatic Annotation Server (http://www.genome.jp/kegg/) were carried out for KEGG annotation and classification^76^. Quantification of gene expression levels was estimated by RSEM (version v1.2.15)^77^. Differential expression analysis between adjacent developmental stages was implemented using the DESeq R package^78^. The absolute value of log_2_Ratio ≥ 1 and false discovery rate (FDR) ≤ 0.001 were used as the threshold to judge the significance of each gene expression difference. The heat maps were grouped together according to their FPKM values by Cluster 3.0^79^ and visualized by TreeView 1.6^80^. Moreover, in the priority order of NR and SwissProt, the best-matched fragments of the annotated unigenes were predicted for coding regions (CDS) using blastx with E value < 0.00001. The the CDS of the remaining un-annotated unigenes were predicted using ESTScan.

### Real-time qPCR amplification for RNA-Seq verification

To verify the accuracy of RNA-seq data, eight DEGs were selected for verification by real-time qPCR amplification in the early development and moulting stages respectively, with gene-specific primers designed by Primer 5.0 (Supplementary Table S9). Myosin light chain (c26953_g1) was used as the control reference gene, and relative gene expression levels were calculated using the comparative Ct method with the formula 2^−ΔΔCt^ ^81^. All samples were run in triplicates in separate tubes and each cDNA sample was run in duplicate (Supplementary Figure S2).

### Data availability

The sequence data in the present study have been released to the NCBI Sequence Read Archive database (http://trace.ncbi.nlm.nih.gov/Traces/sra/sra.cgi?view=studies) and the accession numbers of samples are SRS1145819 and SRS1828174 for two reference transcriptome, SRP094135 for nine early developmental stages and SRP061180 for eight adult moulting stages.

## Electronic supplementary material

Supplementary information

Table S1

Table S2

Table S3_BLASTx

Table S3_ESTScan

Table S4

Table S5

Table S6

Table S7

Table S8

Table S9
