# Supplementary material for: Transcriptome analysis on the exoskeleton formation in early developmetal stages and reconstruction scenario in growth-moulting in Litopenaeus vannamei
Source: Sci Rep. 2017 Apr 24;7:1098. doi: 10.1038/s41598-017-01220-6 (PMC5430884; doi:10.1038/s41598-017-01220-6)
Supplement: Supplementary file 1 — Supplementary information [file 41598_2017_1220_MOESM1_ESM.pdf]

Transcriptome analysis on the exoskeleton formation in early developmetal stages and reconstruction scenario in growth-moulting in *Litopenaeus vannamei*

Yi Gao[1], Jiankai Wei[3], Jianbo Yuan[1], Xiaojun Zhang[1, 2*], Fuhua Li[1, 2] and Jianhai Xiang[1, 2*]

[1] Key Laboratory of Experimental Marine Biology, Institute of Oceanology, Chinese Academy of Sciences, Qingdao 266071, China.

[2] Laboratory for Marine Biology and Biotechnology, Qingdao National Laboratory for Marine Science and Technology, Qingdao 266071, China.

[3] Ocean University of China, Qingdao 266071, China.

[*]Address correspondence to Jianhai Xiang email: jhxiang@qdio.ac.cn; Xiaojun Zhang email: xjzhang@qdio.ac.cn

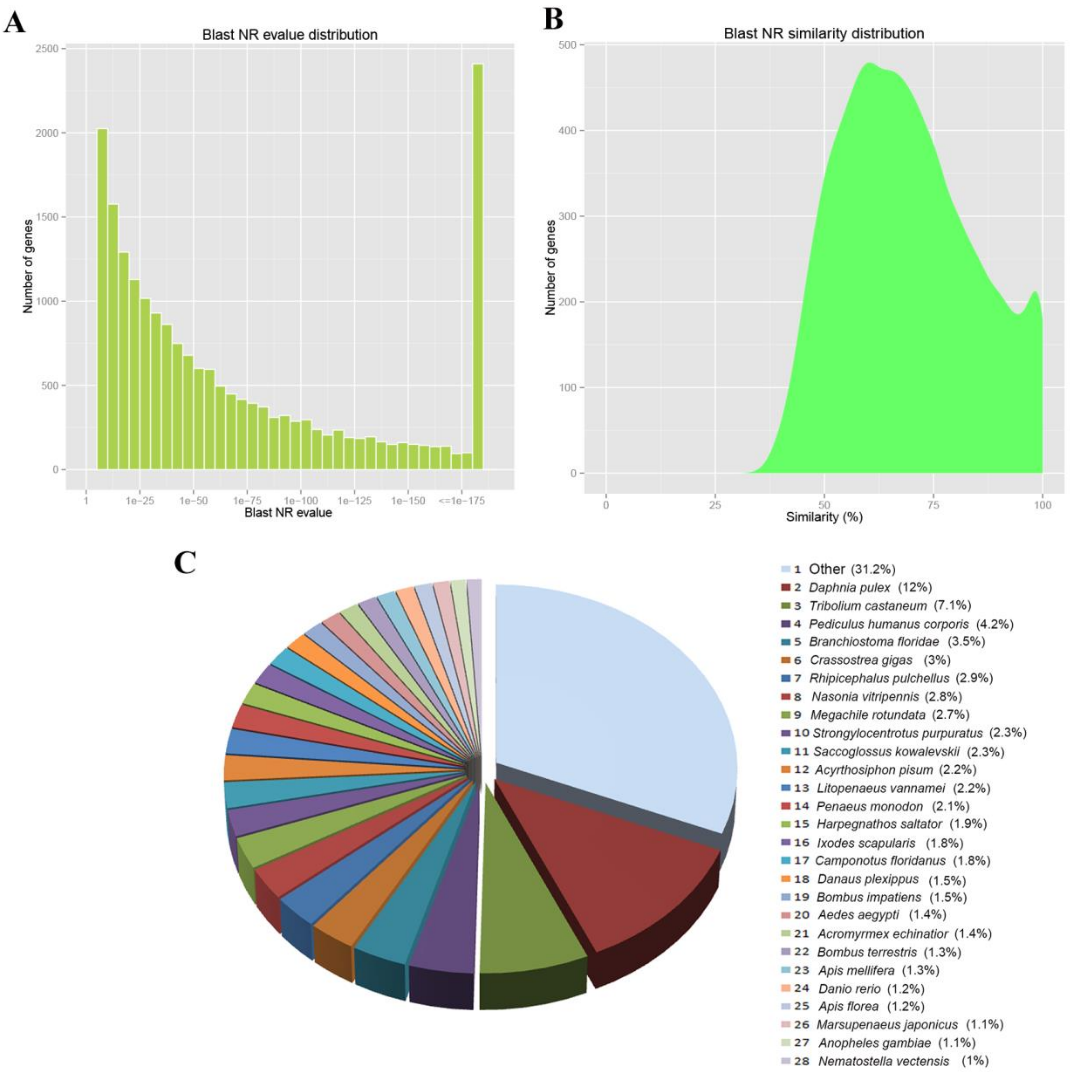

Fig. S1 Summary of the homology search for all unigenes against the NR database. (a) E-value distribution of the best blast hits; (b) Similarity distribution of the best blast hits; (c) Species distribution of the best blast hits.

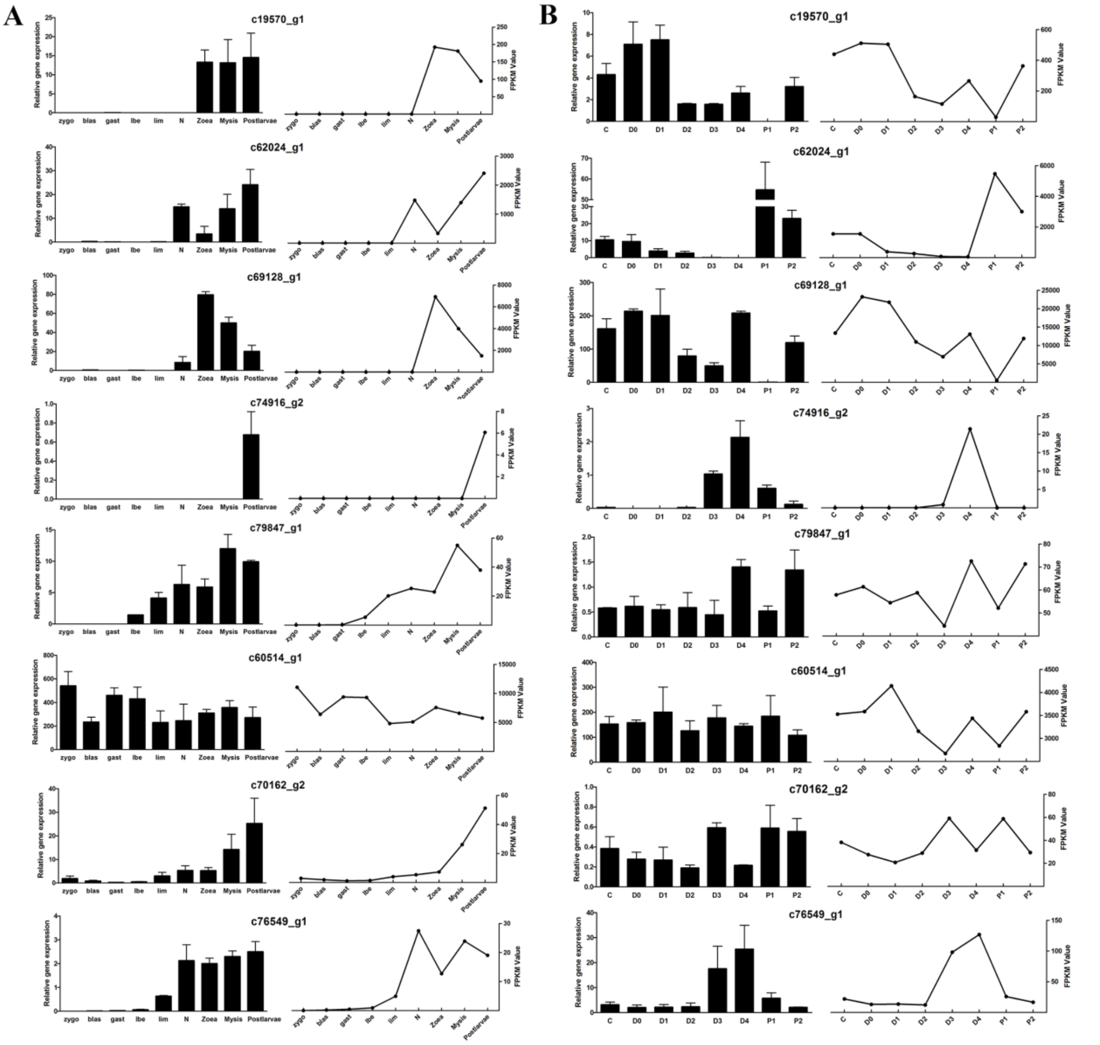

Fig. S2. Real-time quantitative PCR (qRT-PCR) validation. Eight differentially expressed genes were selected for validation in nine early development stages (A) and eight moulting stages (B). Expression results of qRT-PCR are shown on the left and expression profiles of RNA-sequencing on the right. The data are expressed as the mean +SD after normalization.
