# Supplementary material for: Transcriptome analysis on the exoskeleton formation in early developmetal stages and reconstruction scenario in growth-moulting in Litopenaeus vannamei
Source: Sci Rep. 2017 Apr 24;7:1098. doi: 10.1038/s41598-017-01220-6 (PMC5430884; doi:10.1038/s41598-017-01220-6)
Supplement: Supplementary file 10 — Table S8 [file 41598_2017_1220_MOESM10_ESM.docx]

**Table S8** Details of the categories related to the pathway ko04978 “mineral absorption” and ko04961 “endocrine and other factor-regulated calcium reabsorption”. It displays the KO ID, definition, members and expression patterns in moulting.

| Pathway | KO ID | KO Definition | UniGene | Expression pattern |
| --- | --- | --- | --- | --- |
| Mineral absorption (ko04978) | K04982 | transient receptor potential cation channel subfamily M member 7 | c80764_g1 | 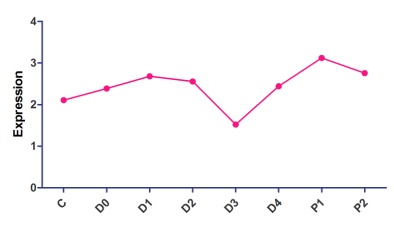 |
|  | K01539 | sodium/potassium-transporting ATPase subunit alpha | c69930_g1 c69930_g2  c75341_g1 | 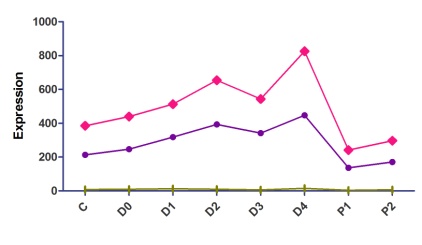 |
|  | K00522 | ferritin heavy chain | c76389_g2  c100000_g1  c68841_g1  c12729_g1  c93718_g1  c129388_g1  c57168_g1  c57597_g1 | 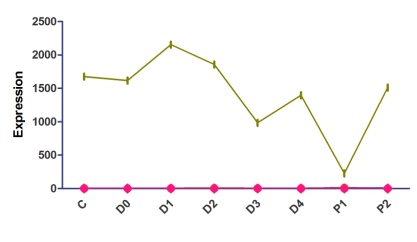 |
|  | K01540 | sodium/potassium-transporting ATPase subunit beta | c86428_g1 c71361_g1 c57094_g2 c84939_g1 | 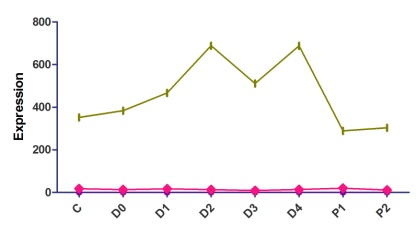 |
|  | K07213 | copper chaperone | c14052_g1 c68977_g1 | 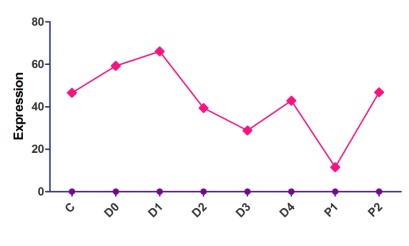 |
|  | K05011 | chloride channel 2 | c82606_g1 c82927_g2 c82927_g1 | 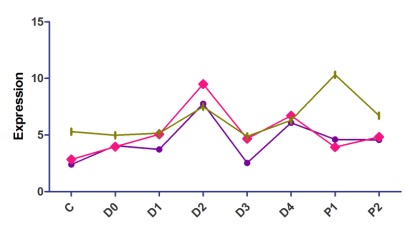 |
|  | K12347 | natural resistance-associated macrophage protein | c67838_g1 | 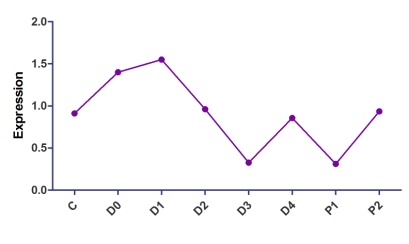 |
|  | K14704 | solute carrier family 26 (sulfate anion transporter), member 6 | c79959_g1 | 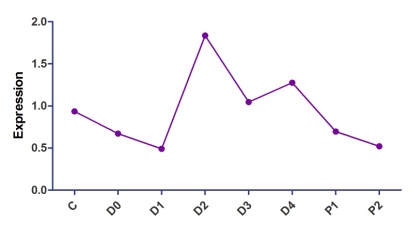 |
|  | K08539 | vitamin D3 receptor | c77829_g2 | 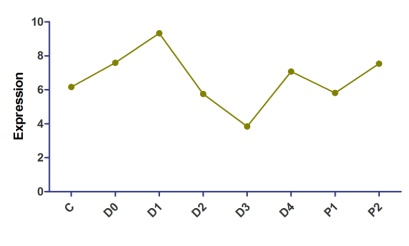 |
|  | K12040 | solute carrier family | c81046_g2 c82850_g3 c78696_g1 c75401_g1 c80370_g1 c76650_g2 | 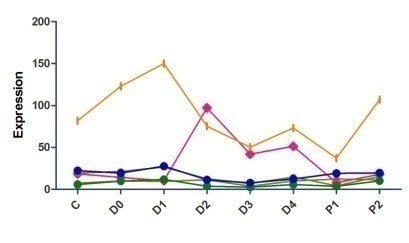 |
|  | K13625 | ferritin light chain | c39486_g2 c39486_g1 | 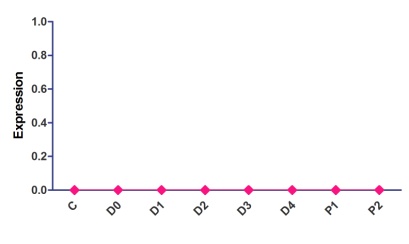 |
| Endocrine and other factor-regulated calcium reabsorption （ko04961） | K04345 | protein kinase A | c79621_g1 c71640_g1 | 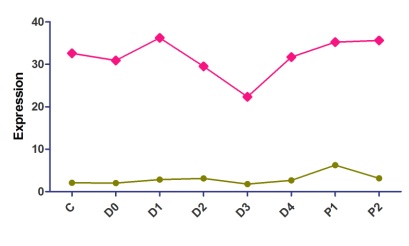 |
|  | K11824 | AP-2 complex subunit alpha | c78755_g2 | 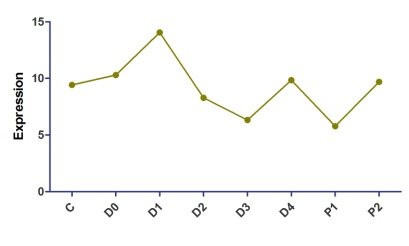 |
|  | K08046 | adenylate cyclase 6 | c75748_g2 | 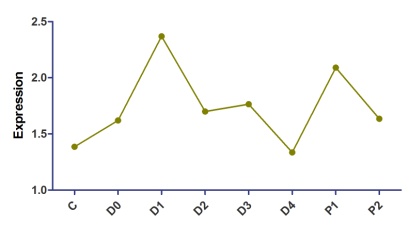 |
|  | K04646 | clathrin heavy chain | c2644_g1 c27548_g1 | 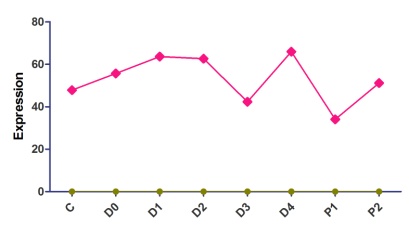 |
|  | K02677 | classical protein kinase C | c78912_g2 | 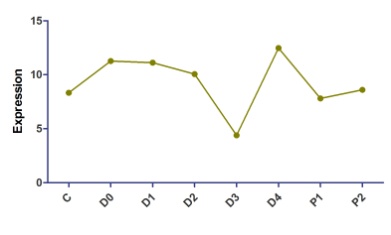 |
|  | K08049 | adenylate cyclase 9 | c80996_g2 c80996_g1 | 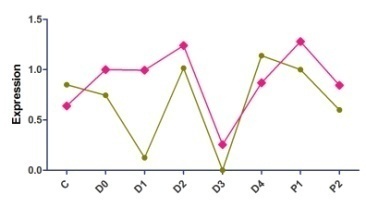 |
|  | K01539 | sodium/potassium-transporting ATPase subunit alpha | c69930_g1 c69930_g2 c75341_g1 c86428_g1 c71361_g1 c57094_g2 c84939_g1 | 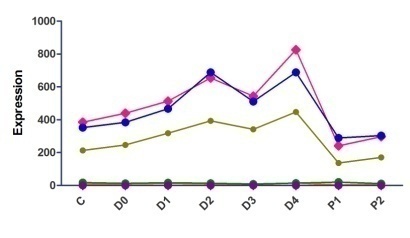 |
|  | K08539 | vitamin D3 receptor | c77829_g2 | 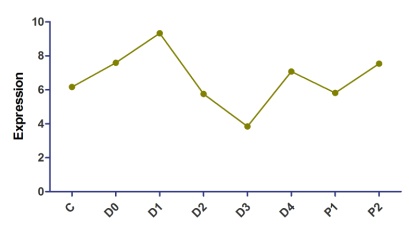 |
|  | K05858 | phosphatidylinositol phospholipase C, beta | c76861_g1 c82038_g1 | 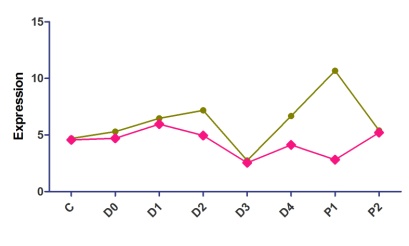 |
|  | K11827 | AP-2 complex subunit sigma-1 | c119408_g1 c59773_g1 | 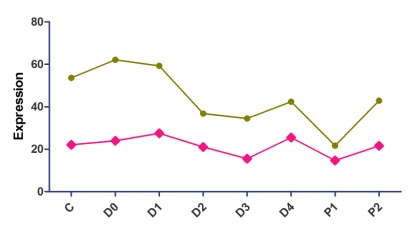 |
|  | K04632 | guanine nucleotide-binding protein G(s) subunit alpha | c71661_g1 c105728_g1 c81857_g1 | 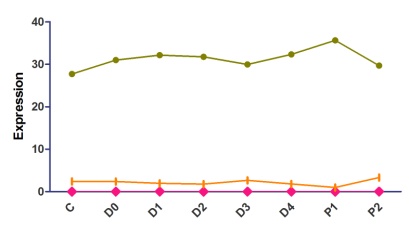 |
|  | K04585 | parathyroid hormone receptor 1 | c41116_g1 | 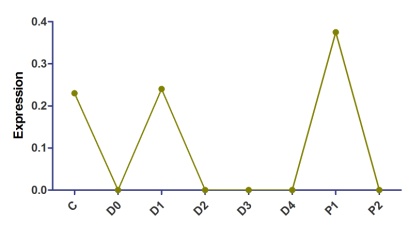 |
|  | K04634 | guanine nucleotide-binding protein G(q) subunit alpha | c42435_g1 | 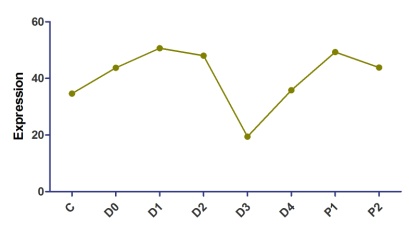 |
|  | K04644 | clathrin light chain A | c71279_g2 | 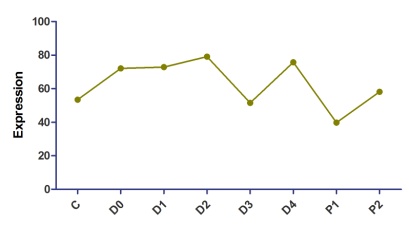 |
|  | K07904 | Ras-related protein Rab-11A | c82512_g2 | 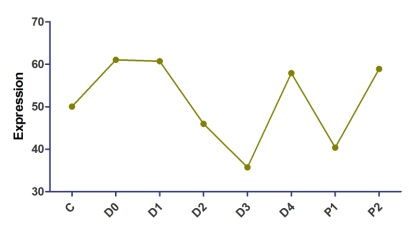 |
|  | K01528 | dynamin GTPase | c79893_g2 c69970_g1 | 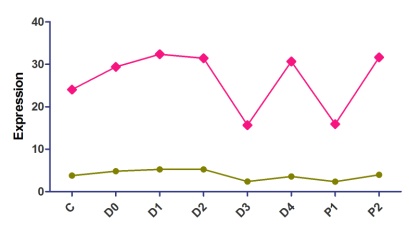 |
