# Supplementary material for: Transcriptome analysis on the exoskeleton formation in early developmetal stages and reconstruction scenario in growth-moulting in Litopenaeus vannamei
Source: Sci Rep. 2017 Apr 24;7:1098. doi: 10.1038/s41598-017-01220-6 (PMC5430884; doi:10.1038/s41598-017-01220-6)
Supplement: Supplementary file 11 — Table S9 [file 41598_2017_1220_MOESM11_ESM.docx]

**Table S9** Primers of eight genes and the reference gene for verification experiment, it indicates the gene description and product length in brackets

| Gene ID and Description | Primer sequence |
| --- | --- |
| C19570_g1（ecdysteroid regulated-like protein, 159bp） | **Sense:** GTCCTTGCCGAATACCCT |
|  | **Anti-sense:** ACAATCCTTCTGGCTGCTC |
| C62024_g1（cuticle protein CUT8, 210bp） | **Sense:** GTCGTGGGAGAACTGGGT |
|  | **Anti-sense:** CGCTCGGGAAAGTATAAAAG |
| C69128_g1（trypsin-like serine proteinase, 157bp） | **Sense:** TCTGCTCGTTGCCCTCATC |
|  | **Anti-sense:** GGCTTCGCCTTCCACTTCT |
| C74916_g2（molting fluid carboxypeptidase A precursor, 215bp） | **Sense:** CCTCACCATTCCGTCTGCT |
|  | **Anti-sense:** ACCCTTGCTCCGACCTCT |
| C79847_g1（chitin synthase 1, 113bp） | **Sense:** TCCGTATTTTTCCGCTTT |
|  | **Anti-sense:** CATACAAGGGAATCCAAGC |
| C60514_g1（cytochrome c oxidase subunit III, 155bp） | **Sense:** GTGACGGGATATTACACG |
|  | **Anti-sense:** TAGGAGCAAGTCTTCTGTG |
| C70162_g2（voltage-dependent calcium channel gamma-2 subunit-like, 157bp） | **Sense:** CGGTTCCACTTGGCACTA |
|  | **Anti-sense:** GATGGTCGGTTCCTCCTC |
| C76549_g1（nuclear receptor E75 protein, 85bp） | **Sense:** GAAGACTTGGTCGTCACAGAG |
|  | **Anti-sense:** CGCCATAGTGGAAACCTG |
| c26953_g1（myosin light chain, 301bp） | **Sense:** GGACGCACTGGCTGACTGT |
|  | **Anti-sense:** CCACCTGCTCGCTCTTACC |
